# Synthesis of Highly Conductive Electrospun Recycled Polyethylene Terephthalate Nanofibers Using the Electroless Deposition Method

**DOI:** 10.3390/nano11020531

**Published:** 2021-02-19

**Authors:** Nadir Hussain, Mujahid Mehdi, Muhammad Yousif, Aizaz Ali, Sana Ullah, Sajid Hussain Siyal, Tanweer Hussain, Ick Soo Kim

**Affiliations:** 1 Nano Fusion Technology Research Group, Division of Frontier Fibers, Institute for Fiber Engineering (IFES), Interdisciplinary Cluster for Cutting Edge Research (ICCER), Shinshu University, Tokida 3-15-1, Ueda, Nagano Prefecture 386-8567, Japan; engr.nadir712@hotmail.com (N.H.); sanamalik269@gmail.com (S.U.); 2 National Engineering Laboratory for Modern Silk, College of Textile and Clothing Engineering, Soochow University, Suzhou 215123, China; mujahid11te83@gmail.com (M.M.); muhammadyousif.te72@gmail.com (M.Y.); 3 State Key Laboratory for Modification of Chemical Fibers and Polymer Materials, College of Materials Science and Engineering, Donghua University, Shanghai 201620, China; aizazali100@gmail.com; 4 Department of Metallurgy and Materials Engineering, Dawood University of Engineering and Technology, Karachi 74800, Pakistan; sajid.hussain@duet.edu.pk; 5 Centre of Excellence in Nanotechnology and Materials, Mehran University of Engineering and Technology, Jamshoro 76060, Pakistan; tanweer.hussain@faculty.muet.edu.pk

**Keywords:** electrospinning, nanofibers, electroless deposition, wearable electronics, waste drinking bottles

## Abstract

Plastic bottles are generally recycled by remolding them into numerous products. In this study, waste from plastic bottles was used to fabricate recycled polyethylene terephthalate (r-PET) nanofibers via the electrospinning technique, and high-performance conductive polyethylene terephthalate nanofibers (r-PET nanofibers) were prepared followed by copper deposition using the electroless deposition (ELD) method. Firstly, the electrospun r-PET nanofibers were chemically modified with silane molecules and polymerized with 2-(methacryloyloxy) ethyl trimethylammonium chloride (METAC) solution. Finally, the copper deposition was achieved on the surface of chemically modified r-PET nanofibers by simple chemical/ion attraction. The water contact angle of r-PET nanofibers, chemically modified r-PET nanofibers, and copper deposited nanofibers were 140°, 80°, and 138°, respectively. The r-PET nanofibers retained their fibrous morphology after copper deposition, and EDX results confirmed the presence of copper on the surface of r-PET nanofibers. XPS was performed to analyze chemical changes before and after copper deposition on r-PET nanofibers. The successful deposition of copper one r-PET nanofibers showed an excellent electrical resistance of 0.1 ohms/cm and good mechanical strength according to ASTM D-638.

## 1. Introduction

Flexible electrically conductive materials have gained significant demand for electronics and telecommunication systems because of their capability to convey electrical properties in this century [1]. The increasing demand of materials for smart electronics that can work under mechanical deformation with high efficiency and the required conductivity has become a high stream/demanding field of research [2]. The polymer and metal composites have been extensively explored [3] with a variety of new applications including flexible displays, wearable electronics, energy devices, biological actuators, and smart electronic skin [4,5,6,7,8]. The parameters of these smart materials can work under mechanical deformation and interconnect with great mechanical flexibility (i.e., bending, stretching, twisting, and compressing) [1].

Wearable electronics rely heavily on metal-coated textiles to produce textile materials with special functional properties. In the previous research studies, textile based conductive materials were widely fabricated via various deposition methods [9,10]. Currently, some advanced techniques have been introduced to fabricate smart conductive textile materials such as atomic layer deposition [11], galvanic deposition, and electroless deposition (ELD) of metals [12,13]. Among the above techniques, ELD is the most utilized technique because of its low cost and easy process of fabrication at room temperature [14,15]. The ELD technique has been extensively explored in many advanced applications including electrically conductive textiles [16], EMI shielding [17,18], telecommunication frequencies, and electrostatic discharge [19]. Generally, the ELD method is carried out in three steps: the functional polymer anchoring layer by surface modification, the filling of catalyst on the polymer anchoring layer with ion exchange, and the deposition of site selective metals. Previously, ELD with different textile fabrics such as polyester, cotton, nylon, and Kevlar showed electrical properties with low cost at room temperature [20,21,22,23].

The previous textile based conductive fabrics contain a low surface area and high porous structure, which can limit the metal deposition rate. Electrospun nanofibers have gained much attraction in wide applications due to their high surface area and can create a high metal deposition rate [2]. Recently, polyester nanofibers have been achieved from waste drinking bottles having good mechanical strength and flexibility [24]. This is an alternate method to produce products from waste material and minimize solid waste in the environment. In 2019, the global market of recycled polyethylene terephthalate (r-PET) was valued at USD 7.34 billion, with a growth projection of 7.9% until 2027. These data show the importance of the r-PET market with consumers being more conscious about environmental sustainability [25].

Nanotechnology has deep roots in every field of life and in helping the research community to solve a wide a range of challenges [26]. Electrospinning is a simple technique to produce fibers with diameters in the range of a few tens to hundreds of nanometers. Electrospun nanofibers have a range of applications such as food packaging, wound dressings, tissue engineering, water filtration [27,28,29,30], air filtration, facemasks, flexible electronics, and sensors [31]. Keeping in the broad scope of electrospun nanofibers, the aim of this study is to investigate the ELD performance on electrospun nanofiber to fabricate flexible and conductive materials. The r-PET nanofibers were fabricated via the electrospinning technique, and copper metal was deposited by following the ELD method. The deposition of copper metal on r-PET nanofibers was characterized using scanning electron microscopy (SEM), energy dispersive X-ray spectroscopy (EDX), X-ray photoelectron spectroscopy (XPS), water contact angle, and tensile strength. Finally, the surface conductivity of metal deposited r-PET nanofibers was assessed using a four-point probe meter.

## 2. Experimental Section

### 2.1. Materials

Recycled polyethylene terephthalate (r-PET) was obtained from water bottles at a convenience store in Japan and were utilized directly without further purification. Trifluoracetic acid, chloroform, vinyltrimethoxysilane (98%), (2-(methacryloyloxy) ethyl) tri-methyl-ammonium chloride solution (METAC), ammonium tetra-chloro-palladate (II), and copper (II) sulfate pentahydrate were purchased from Sigma Aldrich USA (St. Louis, MI, USA). Chemical reagents, potassium sodium tartrate tetrahydrate, formaldehyde, potassium persulfate, and sodium hydroxide were purchased from Sinopharm Chemical Reagent Co., Ltd. (Huangpu, Shanghai, China).

### 2.2. Fabrication of r-PET Nanofibers

The r-PET nanofibers were fabricated by following the previous protocol [24]. Briefly, r-PET bottles were cut into small pieces (approximately 1 × 1 cm^2^) and dissolved in the TFA/chloroform with a ratio of 1:3 in order to make a 15% *w*/*w* polymer composition. Electrospinning was performed using a high-voltage power supply (Har − 100 × 12, Matsusada Co.; Tokyo, Japan) at room temperature. The prepared solution was poured into a syringe with a capillary tip attached having an internal diameter of 0.6 mm and keeping a 0.5 mL/h flow rate with a supplied voltage of 15 kV. The as-prepared r-PET nanofibers were collected on aluminum foil wrapped over a rotary collector system, and the capillary tip to collector distance was set as 15 cm. After electrospinning, the r-PET nanofibers were peeled off and dried overnight before further use.

### 2.3. Fabrication of Conductive r-PET Nanofibers

Graphical abstract represents the complete fabrication process to prepare conductive r-PET nanofibers and characterized by 4 steps (plasma treatment, silanization, polymerization, and electroless deposition). Each step is explained below.

#### 2.3.1. Plasma Treatment

The obtained r-PET nanofibers were hydrophobic, and to create hydroxyl groups before the silanization process, plasma treatment was needed. Previously, plasma treatment was used to create hydroxyl groups on textile substrates [2]. The r-PET nanofibers were treated with air plasma using Harrick Plasma cleaner PDC-002, and the parameters for plasma treatment are listed below in Table 1.

#### 2.3.2. Silanization

The plasma treated r-PET nanofibers were placed in an ethanol solution having 10% (*v*/*v*) vinyl-tri-methoxy-silane (VTMS) for 20 min. The VTMS-treated r-PET nanofibers were rinsed with DI water and immediately vacuum dried at 50 °C. The successful VTMS treatment provided the hydrophobic surface of r-PET nanofibers due to the presence of dense vinyl groups.

#### 2.3.3. Polymerization

The silanized-treated r-PET nanofibers were immersed into a polymerization solution, which contained a mixture of 20% (*v*/*v*) METAC aqueous solution (100 mL) and 60 mg of potassium persulfate (KPS) for 60 min to carry out the polymerization of METAC (PMETAC) brushes while stirring at 60 °C. Finally, the polymerized r-PET nanofibers were washed with DI water and vacuum dried at 50 °C for 2 h.

#### 2.3.4. Electroless Deposition

The electroless deposition (ELD) of copper was carried out on PMETAC r-PET nanofibers by following previous protocols [20,22,23,24]. Briefly, the PMETAC r-PET nanofibers were immersed in a 5 mM aqueous solution of ammonium tetra-chloro-palladate (II) (NH_4_)_2_PdCl_4_) for 20 min for the ion-exchange reaction. During the ion-exchange, [PdCl_4_]^2-^ catalytic ions were immobilized onto the quaternary ammonium groups of PMETAC polymer chains, and the samples were gently washed with DI water. Further, an ELD bath was prepared to contain Solutions A and B (Table 2) with a ratio of 1:1, where Solution A consisted of 13 g/L copper sulfate pentahydrate, 12 g/L sodium hydroxide, and 29 g/L potassium sodium tartrate tetrahydrate, while Solution B contained formaldehyde 9.5 mL/L in DI water. A detailed summary of the chemicals used during the fabrication of the conductive r-PET nanofibers is given in Table 2 and Table 3.

### 2.4. SEM Morphology

Scanning electron microscopy (S-3000N; Hitachi, Tokyo, Japan) with an acceleration voltage of 30 kV was used to observe the surface morphology of the neat r-PET nanofibers and copper-coated r-PET nanofibers. The EDX images and spectrum were obtained using an S-3000 N, Hitachi Ltd. X-ray spectroscopy, Japan. All samples of SEM and EDX were sputter coated with gold before analysis. 

### 2.5. XPS Analysis

The X-ray spectroscopy (XPS) (S-3000 N, Hitachi, Tokyo, Japan) was performed to analyze the chemical composition of the neat r-PET nanofibers and copper-coated r-PET nanofibers.

### 2.6. Water Contact Angle

An OCA-40 contact angle instrument (Data physics Filderstadt Germany) was applied to determine the water contact angle of neat r-PET nanofibers and each process involved to fabricate the conductive r-PET nanofibers.

### 2.7. Tensile Strength Properties

The mechanical properties of neat r-PET nanofibers and copper-coated r-PET nanofibers after the ELD process were tested by a Titan Universal Tester 3-910, Titan Company Ltd., Hessen, Germany. The tensile strength was measured according to the ASTM D-638 standard. During the test, the speed was set at 5.0 mm/min. The values of the stress-strain curves and Young’s modulus were calculated by using the following Equations (1)–(3), respectively [32].

(1) $$\varepsilon =\frac{\Delta l}{l}$$

(2) $$\sigma \;=\;\frac{F}{A}$$

(3) $$E\;=\;\frac{\sigma }{\varepsilon }$$

where ε, *σ*, and E represents the stress, strain, and Young’s modulus, respectively. Δl represent the change in length, and *l* shows the original length of the specimen. F is the applied force on the specimen, and A is the area of the specimen.

### 2.8. Shrinkage Test

The shrinkage test was performed by taking 5 × 5 cm^2^ r-PET nanofibers with an average thickness of 120 µm. The shrinkage of r-PET nanofibers after the ELD process was calculated by using Equation (4) for lengthwise shrinkage and Equation (5) for widthwise shrinkage, respectively.

(4) $$\mathrm{Ls}\;=\;\frac{L-\Delta L}{L}\;\times 100,$$

(5) $$\mathrm{Ws}\;=\;\frac{W-\Delta W}{W}\;\times 100,$$

(6) $$Rs=\sqrt{Ls^{2}+Ws^{2}}$$

In Equation (4), ***Ls*** is lengthwise shrinkage, ***L*** is the original length of the samples, and **∆*L*** is the change in the original length after the ELD process. In Equation (5), ***Ws*** is widthwise shrinkage. ***W*** is the original width of the samples. **∆*w*** is the change in the original width after the ELD process. In Equation (6), ***Rs*** is the residual shrinkage after the ELD process.

## 3. Results and Discussion

### 3.1. Scanning Electron Microscopy

**Figure 1:**
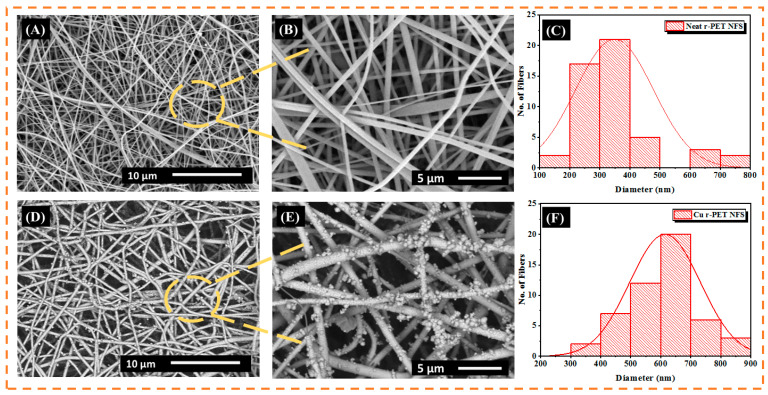
(**A**) SEM image of neat recycled polyethylene terephthalate (r-PET) nanofibers; (**B**) magnified SEM image of neat r-PET nanofibers; (**C**) diameter distribution of neat r-PET nanofibers; (**D**) SEM images of copper-coated r-PET nanofibers; (**E**) magnified SEM image of r-PET nanofibers; and (**F**) diameter distribution of copper-coated r-PET nanofibers.

In this study, neat r-PET nanofiber samples were assessed by SEM (scanning electron microscopy) and analyzed to identify the surface morphology before and after the copper ELD process. The SEM images are shown in Figure 1A, which indicate that neat r-PET nanofibers have a smooth surface area and good morphology with an average diameter of 350 nm, whereas after electroless deposition of the copper, a conformal layer on the r-PET nanofibers was produced while retaining the smooth nanofiber surface texture, as shown in Figure 1C, with an average diameter of 700 nm. The copper-coated r-PET nanofibers had a diameter around 700 nm, which is slightly higher than the pristine r-PET nanofibers due to copper deposition on the nanofibers [24].

### 3.2. EDX Analysis

**Figure 2:**
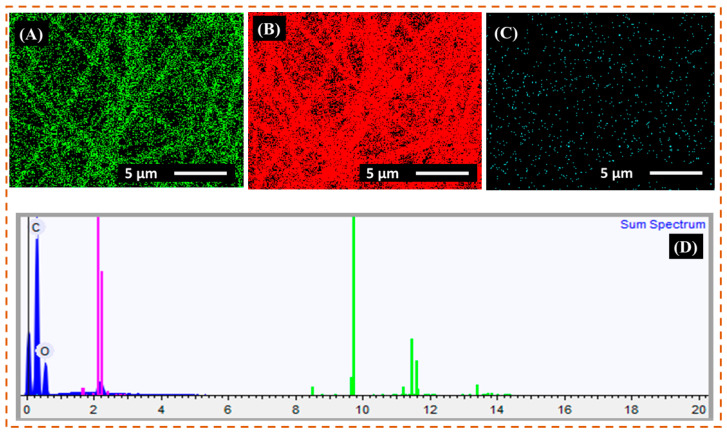
EDX elemental mapping and spectrum of neat r-PET nanofibers. (**A**–**C**) show the oxygen, carbon, and gold elemental mapping, respectively. (**D**) EDX full spectrum of neat r-PET nanofibers.

**Figure 3:**
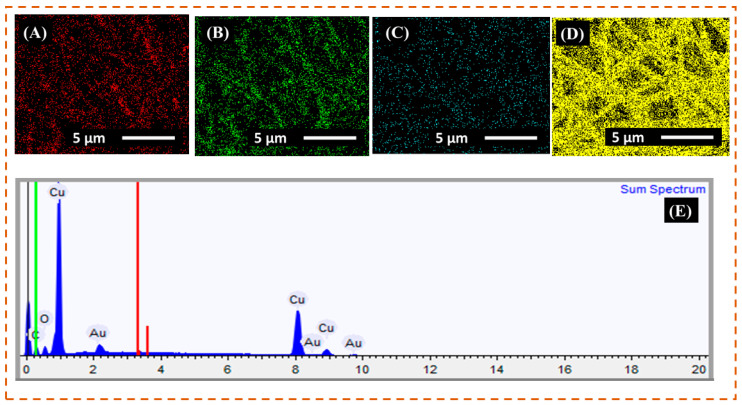
EDX elemental mapping and spectrum of copper-coated r-PET nanofibers. (**A**–**D**) show the oxygen, carbon, gold, and copper elemental mapping, respectively. And (**E**) EDX full spectrum of copper-coated r-PET nanofibers.

The EDX elemental mapping was applied to verify the presence of copper metal in r-PET nanofibers as shown in Figure 2 and Figure 3, respectively. Based on the characterization of the results, the r-PET nanofibers were composed of carbon and oxygen with no other impurities, as shown in Table 4. On the other hand, in Figure 3, the copper-coated r-PET nanofibers were composed of copper, carbon, and oxygen. As presented in Table 5, copper particles were uniformly distributed on the surface of r-PET nanofibers and contained 71% of copper by weight. r-PET nanofibers had a high surface area [33]; therefore, the deposition rate of copper nanoparticles was higher on the copper-coated r-PET nanofibers than the deposition on conventional textile [34].

### 3.3. XPS Analysis

**Figure 4:**
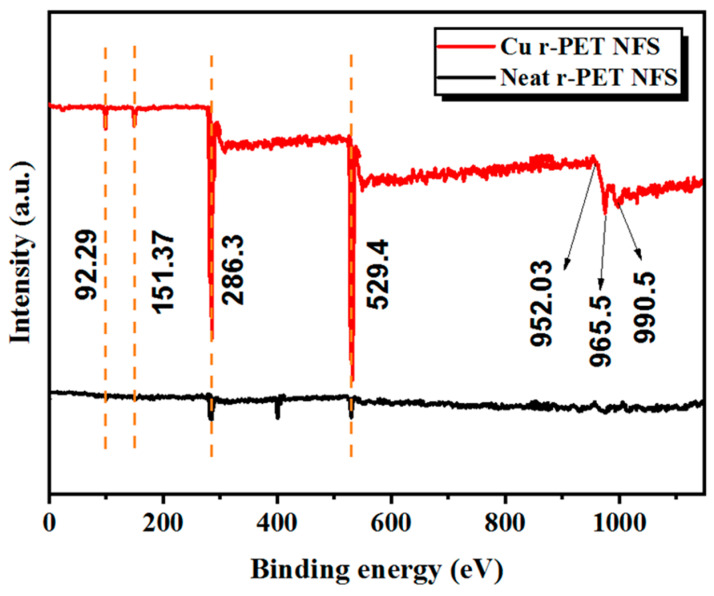
XPS analysis of neat r-PET nanofibers and copper-coated r-PET nanofibers.

To investigate the specific elements and the presence of the chemical bonds, the XPS analysis was performed on neat r-PET nanofibers and copper-coated r-PET nanofibers to ensure the presence of copper elements after the ELD process, which was also confirmed by the EDX process. Figure 4 shows the spectrum peaks with a wide range for all samples of neat r-PET nanofibers and copper-coated r-PET. The XPS spectrum of copper-coated r-PET nanofibers revealed some additional peaks at a binding energy of (990.5–952 eV), which could not be seen in the neat r-PET nanofibers. Furthermore, to ensure the presence of oxygen, nitrogen, sulfur, and carbon groups, along with their bond types, samples were investigated under XPS spectroscopy separately with the respective region and range of the spectrum of the specific elements. Figure 4 shows that the spectrum peaks at a binding energy of (529.4–286.3) in the region O1s, which revealed the same groups of C−O and C = O/O−C = O bonds for neat r-PET nanofibers and copper-coated r-PET nanofibers. As shown in Figure 4, copper-coated r-PET nanofibers revealed additional peaks at a binding energy of (990.5–952 eV) in the region of 2p Cu 2p3/2 Cu 2p1/2, which led to the overlap of copper particles on the surface of nanofibers, which may show the presence of C−C double metallic bonds.

### 3.4. Water Contact Angle Analysis 

**Figure 5:**
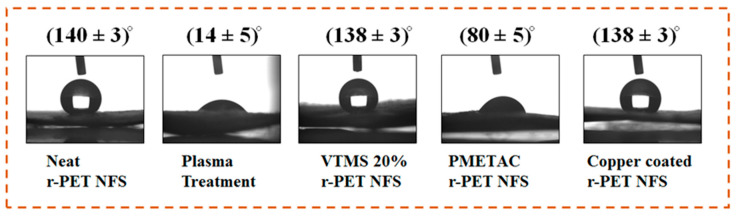
Water contact angle analysis of neat r-PET nanofibers and copper-coated r-PET nanofibers.

The water contact angle was used to examine each successful step required for the ELD process. Figure 5 indicates the water contact angle measurement for each step in the ELD process. The obtained r-PET nanofibers had a hydrophobic nature, and we needed to create hydroxyl groups before the silanization process. Plasma treatment was used to create the hydroxyl groups on the textile substrates, and the water contact angle of r-PET nanofibers changed from (140° ± 5)° to (14 ± 3)° [35]. This change showed that hydroxyl groups were created on the surface of the nanofibers, which were helpful for the VTMS process. r-PET nanofibers were impregnated in a 20% solution of the VTMS in ethanol, which reacted with the silane molecules and hydroxyl groups on the surface of the nanofibers, and the VTMS-treated r-PET nanofibers showed a hydrophobic property, yielding (138 ± 3)° due to the presence of dense vinyl groups, as shown in Figure 5 [36]. Furthermore, the solution was prepared containing the METAC monomer and potassium persulfate (KPS) initiator for polymerization. In the polymerization process, the VTMS based r-PET nanofibers were impregnated for a certain time, and the chemical reaction happened in the meantime. After the polymerization, the water contact angle reached (80 ± 5)°, which might have happened due to the presence of some hydroxyl groups in METAC [37]. In the final process, PMETAC based r-PET nanofibers were impregnated in an ELD bath. After the ELD process, the r-PET nanofibers were fully covered with copper particles, and the water contact angle was (138 ± 5)°, which may be attributed mainly to the presence of copper nanoparticles on the surface of the r-PET nanofibers [20].

### 3.5. Optimum Temperature and Time Study

**Figure 6:**
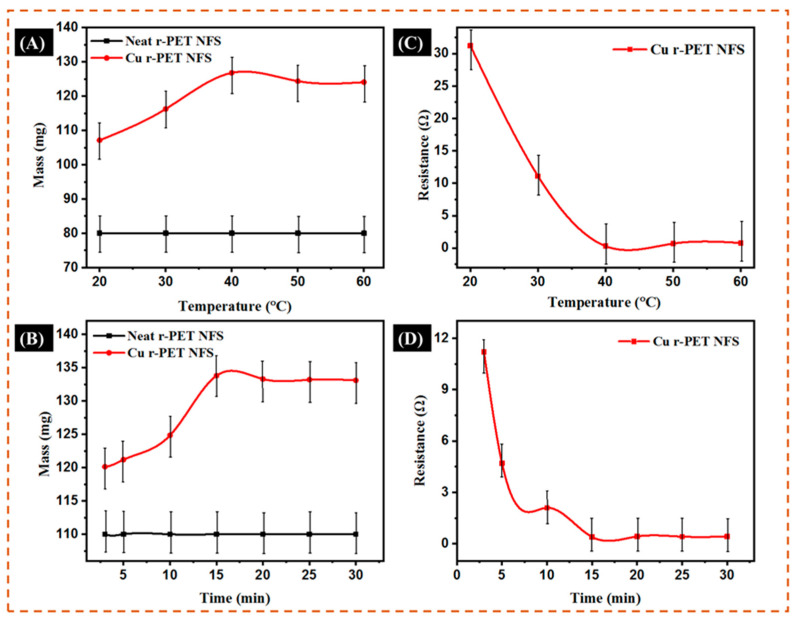
(**A**) The effect of the weight of the r-PET nanofibers as a function of temperature; (**B**) the effect of the resistance of the r-PET nanofibers as a function of temperature; (**C**) the effect of the weight of the r-PET nanofibers as a function of electroless deposition time; (**D**) the effect of the resistance of the r-PET nanofibers as a function of electroless deposition time.

In the electroless deposition, temperature and time had significant effects on the deposition of copper on r-PET nanofibers. Figure 6 shows the results of the samples obtained with different deposition temperatures and times. The temperature for copper deposition on r-PET nanofibers was studied by keeping the time interval constant at 15 min. The effect on the masses at different temperatures in the ELD process is shown in Figure 6A. As the temperature increased, the mass of the r-PET nanofibers also increased and reached a maximum level of rapid increase at 40 °C. Further, with the increase of the temperature, the mass of the r-PET nanofibers was decreased, as shown in Figure 6A. Additionally, the change in the resistance at different temperatures in the ELD process is also shown in Figure 6B. It shows that as temperature increases, resistance decreases and reaches a minimum value at a temperature of 40 °C. By further increasing the temperature, the resistance of the r-PET nanofibers increased, and this might be due to the instability of the solution caused by the higher temperature, which resulted in the poor deposition of the copper [38].

After the optimization of the temperature, for the deposition of copper on the r-PET nanofibers, time optimization was also equally important, as shown in Figure 6C,D. Figure 6C shows a notably higher increase in the deposition of copper from 3 min to 15 min with the deposition rate on the surface of the r-PET nanofibers, which gradually increased up to 30 min. As the deposition time was extended to 30 min, almost all of the surface of the r-PET nanofibers was covered with copper. Hence, in this study, fifteen minutes was the optimum time for the deposition of copper on the r-PET nanofibers. In addition, the mass of the r-PET nanofibers increased as the deposition time was prolonged, while the mass of the r-PET nanofibers remained constant. This might be due to there being no more available sites for copper deposition after 30 min. The plot of the resistance of the copper-coated r-PET nanofibers with different deposition times is shown in Figure 6D. As the deposition time increased, a uniform coating of the metal on the surface of the r-PET nanofibers was formed, which created a conducting path on the surface, and as a result, the resistance decreased. The sample obtained with a deposition time of 15 min showed minimum resistance.

**Figure 7:**
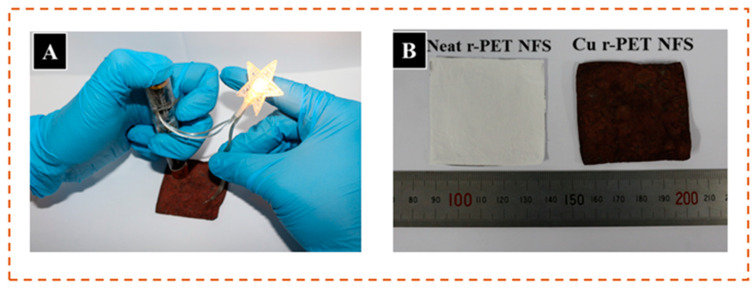
(**A**) Conductivity of r-PET NFS (**B**) results of copper-coated r-PET NFS after the ELD process.

According to the experimental results, continuous copper deposition could be obtained as the deposition time was extended up to 30 min, but the optimal time was observed as 15 min for the deposition of copper on the r-PET nanofibers at a temperature of 40 °C. In the optimum condition, the deposition of copper was good, showing the lowest resistance. Figure 7A,B displays the original photographs of an LED light on the surface of the prepared conductive r-PET nanofibers and the comparison of the r-PET nanofibers after the copper deposition, respectively. These results confirm that the copper was well deposited on the r-PET nanofibers and is highly recommended for use in wearable and flexible electronics applications.

### 3.6. Tensile Strength

**Figure 8:**
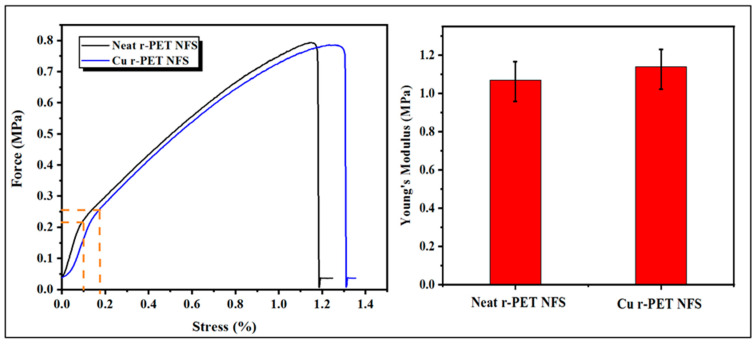
Stress–strain curve and Young’s modulus values of neat r-PET nanofibers and copper-coated r-PET nanofibers after the ELD process.

The mechanical properties of the pristine r-PET nanofibers and copper-coated r-PET nanofibers were investigated. As shown in Figure 8, the tensile strength of pristine r-PET nanofibers was around 1.04 MPa with a deformation of about 1.2%. On the other hand, the tensile strength of copper-coated r-PET nanofibers was around 1.4 MPa with a deformation of about 1.3%. These results depict that there was stronger bonding between the copper particles and r-PET nanofibers. Young’s modulus (elastic modulus) was calculated considering elastic regions of both types of nanofibrous mats. It was observed that the elastic modulus of Cu r-PET nanofibers was slightly increased. This may be because of the presence of metallic nanoparticles (Cu NPs).

### 3.7. Shrinkage Study

The r-PET nanofibers after being exposed to the salinization and the ELD process substantially shrunk, so it was necessary to analyze the shrinkage behavior in the r-PET nanofibers after the ELD process; there was exaggeration in documenting the finding. Table 6 shows the shrinkage results, which show that the shrinkage behavior was also dependent on the medium used irrespective of the ELD process. In copper-coated r-PET nanofibers, the shrinkage percentage was higher than in the neat r-PET nanofibers. Salinization and temperature caused a maximum shrinkage within the copper-coated r-PET nanofibers after the ELD process. Ultimately, shrinkage was also the reason for the greater strength and attachment of the copper particles on the substrate compared to the neat r-PET nanofibers.

## 4. Conclusions

In this research study, copper-coated r-PET nanofibers were successfully fabricated by the process of electroless deposition. The optimized temperature was observed as 40 °C, and the optimized time of 15 min was set for proper copper deposition on the r-PET nanofibers. The SEM images evidently showed the fibrous morphology of the r-PET nanofibers with an average diameter of 350 nm, and the copper-coated r-PET nanofibers retained the fibrous morphology with a slight increase in the average diameter of 700 nm. The optimized copper-coated r-PET nanofibers were composed of 71.6% copper and showed a low electrical resistance of 0.1 Ω. Moreover, the r-PET nanofibers were flexible and showed good mechanical strength. As-prepared, conductive r-PET nanofibers have good potential and could be used in many applications such as wearable electronics, flexible sensors, and energy storage.

## Figures and Tables

**Table 1:** Plasma treatment parameters.

Gas	Pressure (Pa)	Power (W)	Time (min)
Oxygen	30	45	5

**Table 2:** Chemicals used for functionalization. VTMS, vinyl-tri-methoxy-silane; METAC, (2-(methacryloyloxy) ethyl) tri-methyl-ammonium chloride solution.

Chemicals	Formula	Process	Concentration
VTMS	C_5_H_12_O_3_Si	Salinization	20% (*v*/*v* in ethanol)
METAC	C_9_H_18_ClNO_2_	Polymerization	20%
Potassium persulfate	K_2_S_2_O_8_	Ion Exchanger	60 mg/100 mL
Ammonium tetrachloropalladate(II)	(NH4)2PdCl4		5 mM

**Table 3:** Chemicals used for functionalization.

	Chemicals	Formula	Concentration
Solution A	Potassium sodium tartrate	KNaC_4_H_4_O_6_·4H_2_O	29 g/L
	tetrahydrate	NaOH	12 g/L
	Sodium hydroxide		
Solution B	Copper(II) sulfate pentahydrate	CuSO_4_·5H_2_O	13 g/L
	Formaldehyde	HCHO	9.5 g/L

**Table 4:** Summary if the results of elemental mapping.

Element	Weight%	Weight% σ	Atomic%
Carbon	66.649	0.286	72.692
Oxygen	33.351	0.286	27.308

**Table 5:** Summary of the results of elemental mapping.

Element	Weight%	Weight% σ	Atomic%
Carbon	16.098	0.352	46.818
Oxygen	5.786	0.158	12.633
Copper	71.685	0.379	39.409
Gold	6.430	0.242	1.140

**Table 6:** Shrinkage of the r-PET nanofibers after the ELD process.

Shrinkage ^a^	Lengthwise	Widthwise
Cu r-PET NFS	11%	12%

^a^ The shrinkage study was calculated by using Equations (4) and (5).

## Data Availability

The data can be requested from the corresponding author of the article.
